# Supplementary figures and images for: The Protective Effect of Chlorogenic Acid on Vascular Senescence via the Nrf2/HO-1 Pathway
Source: Int J Mol Sci. 2020 Jun 25;21(12):4527. doi: 10.3390/ijms21124527 (PMC7350250; doi:10.3390/ijms21124527)

Figure S1

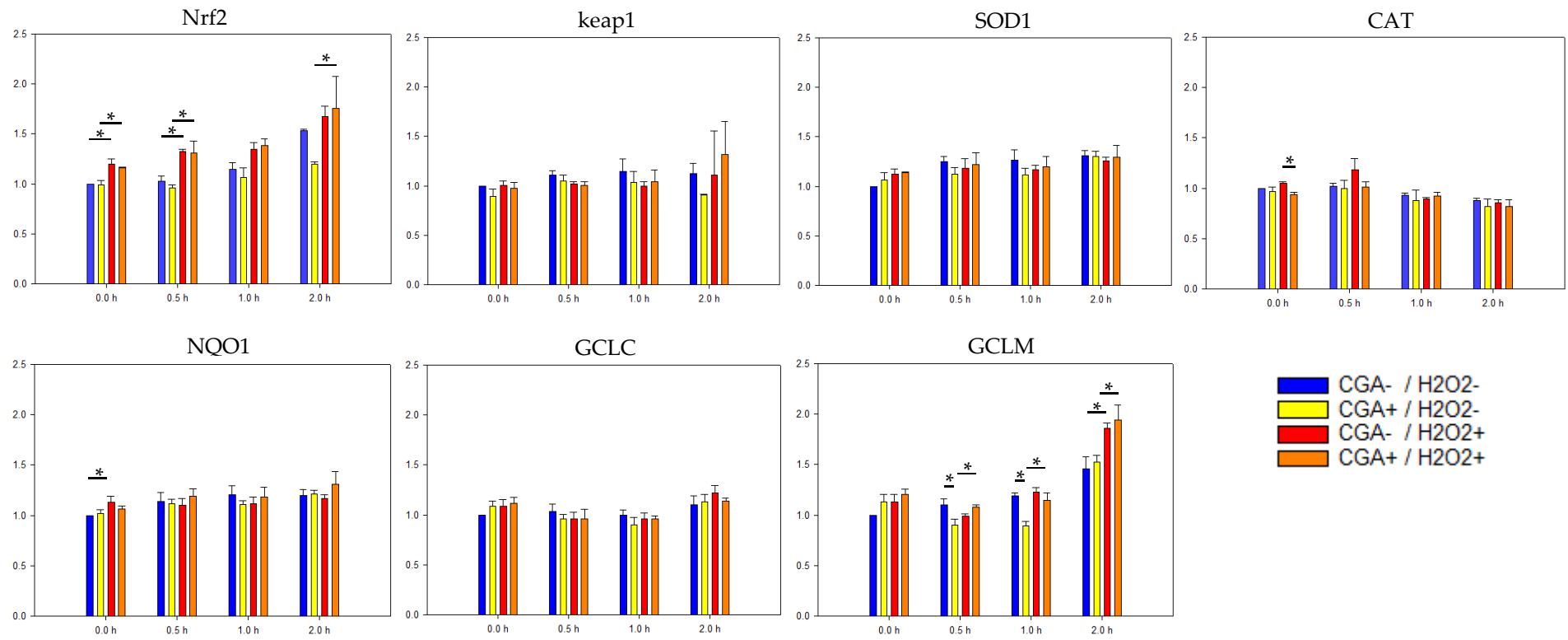

Supplement: Supplementary file 1 [file ijms-21-04527-s001.pdf]
